# National medical specialty guidelines of HIV indicator conditions in Europe lack adequate HIV testing recommendations: a systematic guideline review

**DOI:** 10.2807/1560-7917.ES.2022.27.48.2200338

**Published:** 2022-12-01

**Authors:** Carlijn C.E. Jordans, Marta Vasylyev, Caroline Rae, Marie Louise Jakobsen, Anna Vassilenko, Nicolas Dauby, Anne Louise Grevsen, Stine Finne Jakobsen, Anne Raahauge, Karen Champenois, Emmanuelle Papot, Jakob J. Malin, T. Sonia Boender, Georg M.N Behrens, Henning Gruell, Anja Neumann, Christoph D. Spinner, Frederik Valbert, Karolina Akinosoglou, Evangelia G. Kostaki, Silvia Nozza, Andrea Giacomelli, Giuseppe Lapadula, Maria Mazzitelli, Carlo Torti, Raimonda Matulionyte, Elzbieta Matulyte, Berend J. Van Welzen, Kathryn S. Hensley, Magdalena Thompson, Magdalena Ankiersztejn-Bartczak, Agata Skrzat-Klapaczyńska, Oana Săndulescu, Adrian Streinu-Cercel, Anca Streinu-Cercel, Viktor Daniel Miron, Anastasia Pokrovskaya, Anna Hachfeld, Antonina Dorokhina, Maryna Sukach, Emily Lord, Ann K. Sullivan, Casper Rokx, on behalf of the Guidelines Review Group for the projects: Optimising testing and linkage to care for HIV across Europe (OptTEST by HiE) and the Joint Action on integrating prevention, testing and linkage to care strategies across HIV, viral hepatitis, TB and STIs in Europe (INTEGRATE)

**Affiliations:** 1 Erasmus University Medical Center, Department of Internal Medicine and Department of Medical Microbiology and Infectious Diseases, Rotterdam, the Netherlands; 2 Astar Medical Center, Lviv, Ukraine; 3 Chelsea and Westminster Hospital NHS Foundation Trust, London, United Kingdom; 4 Centre of Excellence for Health, Immunity & Infections (CHIP), Rigshospitalet, University of Copenhagen, Copenhagen, Denmark; 5 Republican Scientific and Practical Center of Medical Technologies, Informatization, Management and Economics of Public Health, Minsk, Belarus; 6 CHU Saint-Pierre, Université Libre de Bruxelles (ULB), and School of Public Health, Université Libre de Bruxelles (ULB), Brussels, Belgium; 7 Université de Paris, INSERM IAME, Paris, France; 8 University of Cologne, Faculty of Medicine and University Hospital Cologne, Department I of Internal Medicine, Division of Infectious Diseases, Cologne, Germany; 9 Robert Koch Institute, Department of Infectious Disease Epidemiology, Berlin, Germany; 10 ECDC Fellowship Programme, Field Epidemiology path (EPIET), European Centre for Disease Prevention and Control (ECDC), Solna, Sweden; 11 Hannover Medical School, Department for Rheumatology and Immunology, Hannover, Germany; 12 Institute of Virology, Faculty of Medicine and University Hospital Cologne, University of Cologne, Cologne, Germany; 13 Institute for Health Care Management and Research, University of Duisburg-Essen, Essen, Germany; 14 Technical University of Munich, School of Medicine, University hospital rechts der Isar, Department of Internal Medicine II, Munich, Germany; 15 Department of Internal Medicine and Infectious Diseases, University General Hospital of Patras, Patras, Greece; 16 Department of Hygiene, Epidemiology and Medical Statistics, Medical School, National and Kapodistrian University of Athens, Athens, Greece; 17 Ospedale San Raffaele, Milano, Italy; 18 III Infectious Diseases Unit, ASST Fatebenefratelli Sacco, Via G.B. Grassi, Milan, Italy; 19 School of Medicine and Surgery, University of Milano-Bicocca, Monza, Italy; 20 Magna Graecia University of Cantanzaro, Catanzaro, Italy; 21 Infectious and Tropical Diseases Unit, University Hospital, Padua, Italy; 22 University Magna Graecia of Catanzaro, Catanzaro, Italy; 23 Department of Infectious Diseases and Dermatovenerology, Faculty of Medicine, Vilnius University; Vilnius University Hospital Santaros Klinikos, Vilnius, Lithuania; 24 Department of Internal Medicine and Infectious Diseases, University Medical Center Utrecht, Utrecht, Netherlands; 25 Hospital for Infectious Diseases, Warsaw, Poland; 26 Foundation of Social Education (FES), Warsaw, Poland; 27 Hospital for Infectious Diseases Warsaw, Medical University of Warsaw, Department of Adults’ Infectious Diseases, Warsaw, Poland; 28 Carol Davila University of Medicine and Pharmacy, Bucharest, Romania; 29 National Institute for Infectious Diseases “Prof.Dr. Matei Bals”, Bucharest, Romania; 30 Central Research Institute of Epidemiology of Rospotrebnadzor, Moscow, Russian Federation; 31 Department of Infectious Diseases, Bern University Hospital and University of Bern, Bern, Switzerland; 32 National Children's Specialized Hospital “OKHMATDYT” of Ministry of Health of Ukraine, Kyiv, Ukraine; 33 O.O.Bogomolets’ National Medical University, Kyiv, Ukraine; 34 Oxford University Hospitals NHS Trust, Oxford, United Kingdom; 35 https://www.opttest.eu/About and https://integrateja.eu/

**Keywords:** HIV, indicator conditions, AIDS-defining conditions, HIV testing recommendations, guidelines, Europe

## Abstract

**Background:**

Adequate identification and testing of people at risk for HIV is fundamental for the HIV care continuum. A key strategy to improve timely testing is HIV indicator condition (IC) guided testing.

**Aim:**

To evaluate the uptake of HIV testing recommendations in HIV IC-specific guidelines in European countries.

**Methods:**

Between 2019 and 2021, European HIV experts reviewed guideline databases to identify all national guidelines of 62 HIV ICs. The proportion of HIV IC guidelines recommending HIV testing was reported, stratified by subgroup (HIV IC, country, eastern/western Europe, achievement of 90–90–90 goals and medical specialty).

**Results:**

Of 30 invited European countries, 15 participated. A total of 791 HIV IC guidelines were identified: median 47 (IQR: 38–68) per country. Association with HIV was reported in 69% (545/791) of the guidelines, and 46% (366/791) recommended HIV testing, while 42% (101/242) of the AIDS-defining conditions recommended HIV testing. HIV testing recommendations were observed more frequently in guidelines in eastern (53%) than western (42%) European countries and in countries yet to achieve the 90–90–90 goals (52%) compared to those that had (38%). The medical specialties internal medicine, neurology/neurosurgery, ophthalmology, pulmonology and gynaecology/obstetrics had an HIV testing recommendation uptake below the 46% average. None of the 62 HIV ICs, countries or medical specialties had 100% accurate testing recommendation coverage in all their available HIV IC guidelines.

**Conclusion:**

Fewer than half the HIV IC guidelines recommended HIV testing. This signals an insufficient adoption of this recommendation in non-HIV specialty guidelines across Europe.

**Key public health message**

**What did you want to address in this study?**

Many people are diagnosed with HIV years after initial infection, often with AIDS. A key strategy for a more timely diagnosis and linking to care is to adequately test people with medical conditions indicative of an underlying HIV infection. This study looked at national clinical practice guidelines throughout Europe to determine what current HIV testing recommendations are given.

**What have we learnt from this study?**

Fewer than half of the HIV indicator condition specific guidelines in Europe contain HIV testing recommendations. Guidelines for medical conditions known to be AIDS defining perform even worse. This deficiency is visible throughout Europe and across all medical specialties. Disturbingly, none of the 62 HIV indicator conditions, countries, or medical specialties have a fully adequate HIV testing recommendation coverage.

**What are the implications of your findings for public health?**

Clinical guidelines define medical practice in national healthcare systems. The omissions we found mean that people with HIV indicator conditions, remain untested. This is a missed opportunity to help healthcare professionals to provide optimal care for a broad range of patients, hinders efforts to stop the spread of HIV through a timely HIV diagnosis, and signals the need to improve national guidelines.

## Introduction

Adequate identification and testing of people at risk for human immunodeficiency virus (HIV) is fundamental for the HIV care continuum. A timely diagnosis of HIV triggers linking people to care, and access to treatment, thus preventing transmission and improving individual health. In 2014, The Joint United Nations Programme on HIV/AIDS (UNAIDS) set the 90–90–90 goals to end the acquired immunodeficiency syndrome (AIDS) epidemic. This cascade of care represents the proportions of people that, by 2020, should be aware of their HIV status, on treatment, and virally suppressed, respectively. Achieving these goals facilitates the desired target of ending the HIV/AIDS epidemic by 2030 [1].

Unfortunately, as of 2020, the 90–90–90 goals had not been achieved in many European countries [2]. Some hopeful signals are however present. Compared to the period 2012 to 2015, the estimated proportion of the total number of people living with HIV (PLWH) who are undiagnosed were found to decrease in eastern Europe from 37% to 18% in 2020 [2]. In western Europe, the proportion of people with undiagnosed HIV decreased to 10% [3,4]. Consequently, the yearly number of new HIV diagnoses in the World Health Organization (WHO) defined European Region increased ca 10% to 136,449 from 2010 to 2019. The increase in new HIV diagnoses was mostly driven by eastern European cases, which also included more AIDS diagnoses than in western Europe. Overall, more than half of newly diagnosed persons with HIV in Europe present with advanced cellular immunodeficiency (CD4^+^ T-cells < 350 cells/µL), and 31% of all new HIV diagnoses are in the AIDS clinical stage [5]. Late presentations and delayed diagnoses increase morbidity and mortality, increase healthcare costs and fuel ongoing HIV transmission [6-8].

A key strategy, endorsed by the WHO and European Centre for Disease Prevention and Control (ECDC), to facilitate timely diagnosis is HIV indicator condition (IC) guided testing. It has been firmly established that HIV IC-guided testing is a cost-effective strategy to find HIV in conditions with an undiagnosed HIV prevalence ≥ 0·1% [9,10]. HIV ICs are also defined as those conditions where not identifying the presence of an HIV infection may have significant adverse health implications [11]. The HIV indicator Diseases across Europe Studies (HIDES) showed the effectiveness of this testing strategy in various settings throughout Europe [10,12]. Despite healthcare professionals regularly encountering patients with HIV ICs, HIV testing rates remain low [5]. Interventions increased HIV testing rates only temporarily with frequent regression to pre-interventional testing rates [13]. A more durable strategy is needed to ensure a widespread implementation of HIV IC-guided testing. As illustrated in other fields of medicine [14-16], national guideline recommendations from medical specialty societies are considered the standards of care and can have sustainable impact on clinical practices. Having HIV testing recommendations implemented universally in HIV IC guidelines should therefore have significant impact on clinical practice.

In light of the high rate of late presentation with HIV in Europe, the aim of this study was to determine the uptake of HIV testing recommendations in HIV IC-specific guidelines of all relevant medical specialties across countries in Europe. The identification of significant gaps would provide opportunities to assimilate HIV testing recommendations and change practice across medical disciplines.

## Methods

### Protocol

A systematic guideline review was conducted to analyse the uptake of HIV testing recommendations in HIV IC guidelines of European countries [17], and followed the Preferred Reporting Items for Systematic Reviews and Meta-Analyses (PRISMA) guidelines [18]. We developed a methodology [19], which was evaluated in a pilot study [20], using a predefined standard operating procedure (the standard operating procedure can be found in Supplement S1). This procedure was then disseminated to participating countries within the established infrastructure of the Optimising Testing and Linkage to Care for HIV across Europe (OptTEST) project [21].

### Data sources and synthesis

The ECDC HIV IC guidance [11] was used to develop a list of 62 HIV ICs, including 25 AIDS-defining conditions (ADCs) and 37 non-AIDS-defining HIV ICs (a list of all ADCs and non-AIDS-defining HIV ICs can be found in Supplementary Table S1). One HIV IC (conditions requiring aggressive immune-suppressive therapy) was excluded from the analysis because it lacked a uniform definition leaving room for subjectivity.

Between 2019 and 2021, we approached HIV expert epidemiologists and medical specialists from 30 European countries affiliated to OptTEST. These experts were invited to participate in the current study via email, and reminders were sent upon non-response. A data lock was set in January 2022, which defined the final dataset used for analysis. The experts were asked to identify all relevant medical specialty guidelines for each ADC and non-AIDS-defining HIV IC by reviewing national guideline databases, national scientific medical specialty society websites, and national primary care physician guidelines. Search engines to identify guidelines used free and medical subject heading (MeSH) search terms, and keywords representing the specific HIV IC and guideline as queries (all databases and references used can be found in Supplement S2). Review instructions were to use all sources and register all identified guidelines, excluding duplicate findings. A negative search result was assumed to reflect a lack of guidelines for this HIV IC. Selected guidelines needed to be endorsed by a medical specialty society. As a reference from outside Europe, available HIV IC guidelines from the WHO on recommending HIV testing were reviewed.

The most up-to-date versions of HIV IC guidelines were used. Each guideline was reviewed by standardised study record forms (the standardised study record form can be found in Supplement S3). Reviewers classified guidelines as: (i) HIV not referenced in the guideline; (ii) association with HIV reported, but HIV testing not recommended; or (iii) association with HIV reported and HIV testing recommended [20]. Inter-observer agreement was checked by two inter-reviewer independent evaluations of all Dutch medical specialty guidelines and appeared sufficient with similar interpretations in 60 of 62 HIV ICs. Each study record form was collected centrally to evaluate missing data, inconsistencies or erroneous entries independently by three investigators (CRa, MLJ, CCEJ). Discrepancies were discussed with three other investigators (AS, CRo, MV). Queries were sent out to reviewers if considered necessary.

To obtain demographics of the HIV epidemic in the participating countries, the most recent available ECDC data were used [5,22-26]. We further stratified the outcomes based on region, UNAIDS 90–90–90 goals and medical specialty. Countries were geographically grouped into two regions according to the WHO European Region definition of western Europe (Belgium, Denmark, France, Germany, Greece, Italy, the Netherlands, Switzerland and the United Kingdom (UK)) and eastern Europe (Belarus, Lithuania, Poland, Romania, Russia and Ukraine). Countries were stratified based on whether they have achieved the UNAIDS 90–90–90 goals (Belgium, Denmark, the Netherlands, Switzerland and the UK) or not (Belarus, France, Germany, Greece, Italy, Lithuania, Poland, Romania, Russia and Ukraine) according to the latest ECDC country report (August 2021). Where no 2020 data were available, the most recent available data were used [2]. Additionally, HIV ICs were categorised according to the following medical specialties: dermatology/venereology, gastroenterology/hepatology, gynaecology/obstetrics, haematology, internal medicine, neurology/neurosurgery, ophthalmology, and pulmonology. Where a condition was treated by multiple specialties or different specialties between countries, a categorisation was performed according to the responsible specialty for the HIV IC in the Netherlands, or, if still unclear, according to the primary organ system affected (e.g. anal carcinoma belongs to gastroenterology) (All HIV ICs grouped per specialty can be found in Supplementary Table S2).

### Data analysis

The primary outcomes were the proportion of HIV IC guidelines reporting a general association of the IC with HIV and the proportion explicitly recommending HIV testing. For the secondary outcomes, we evaluated outcomes according to relevant subgroups by country, eastern/western Europe, achievement of 90–90–90 goals, and per HIV IC or medical specialty. Finally, we developed HIV guideline covering cascades per country where we used three pillars: (i) the proportion of HIV ICs with at least one guideline available; (ii) of the HIV ICs with at least one guideline available, the proportion of at least one guideline available that mentions the relationship with HIV; and (iii) of the HIV ICs with at least one guideline available, the proportion with at least one guideline available that recommends HIV testing. Descriptive statistics are reported as number (percent) and median (interquartile range (IQR)). The associations of the HIV testing recommendations uptake and AIDS-defining status, setting and year of guideline publication was evaluated using chi-squared test. Data from the study record forms were tabulated and aggregated in a Microsoft Excel spreadsheet (Microsoft, Redmond, the United States (US)). Statistical analysis was conducted using SPSS version 25 (IBM, Armonk, US). A p value of ≤0.05 was considered statistically significant.

## Results

### Participating countries and available HIV indicator condition guidelines

Of the 30 European countries invited to take part, 15 participated and sent data before the data lock, including 10 where 90–90–90 goals were not yet achieved (Table 1). In 2020, these 15 countries had an accumulated estimated 1.9 million PLWH (83% of the total within the WHO European Region), of whom 336,000 were estimated to be undiagnosed (18%) [2]. A total of 791 relevant guidelines were identified, with a median number of guidelines per country of 47 (IQR: 38–68). The median number of guidelines per HIV IC was one (range: 0–15), covering ADCs (median 14, IQR: 11–20) and non-AIDS-defining HIV ICs (median 32, IQR: 24–45).

**Table 1:** Number of identified guidelines, number of reviewers, year of guideline publication, 90–90–90 goals and late HIV diagnosis per participating European country, 2019–2021 (n = 15)

Country	Number of identified guidelines	Number of reviewers	Year of guideline publication, median (IQR)	Estimated total people living with HIV^a^	People diagnosed with HIV^a^		People diagnosed with HIV on ART^a^		People on ART who are virally suppressed^a^		Number of cases with CD4^+^ T-cell count^b^		People with late HIV diagnosis in 2020^c^ (CD4^+^ T-cells < 350 cells /µL)^d^	
					n	%	n	%	n	%	n	%	n	%
**Western Europe**														
Belgium	31	1	2013 (2012–2017)	18,335	16,594	91	15,238	92	14,299	94	500	70	203	41
Denmark	83	4	2019 (2018–2020)	6,750	6,150	91	5,670	92	5,550	98	96	89	58	61
France	65	2	2013 (2009–2016)	172,700	148,746	86	133,400	90	126,800	95	1,972	58	1,025	52
Germany	61	7	2018 (2014–2019)	87,900	77,300	88	71,400	92	68,000	88	732	30	383	52
Greece	41	2	2015 (2015–2016)	15,980	13,345	84	10,618	80	NA	NA	423	71	241	51
Italy	38	5	2015 (2012–2018)	130,000	124,500	96	117,000	94	102,000	87	1,223	94	734	60
The Netherlands	71	3	2017 (2014–2018)	23,300	21,360	92	19,913	93	19,046	96	371	94	190	51
Switzerland	34	1	2018 (2016–2019)	16,700	15,500	93	15,000	97	14,800	99	168	59	88	52
United Kingdom	79	3	2011 (2009–2013)	103,800	96,142	93	93,384	97	90,583	97	2,408	87	1,005	42
**Eastern Europe**														
Belarus	47	1	2018 (2012–2018)	26,000	22,084	85	11,714	80	13,575	77	1,105	78	398	36
Poland	37	3	2016 (2013–2018)	15,166	12,385	82	10,496	85	10,052	96	NA	NA	NA	NA
Lithuania	52	2	2015 (2014–2017)	3,397	2,827	77	1,223	43	920	75	NA	NA	NA	NA
Romania	29	4	2014 (2010–2015)	18,000	16,486	92	12,644	77	8,064	64	407	93	228	56
Russia	44	1	2016 (2014–2017)	998,525	808,823	81	319,613	40	271,671	85	57,071	96	16,150	27
Ukraine	76	3	2016 (2007–2016)	251,168	169,787	58	136,105	80	127,871	94	13,791	89	7,513	55

ART: antiretroviral therapy; IQR: interquartile range; NA: not available.

^a^ Data from Continuum of HIV Care, monitoring implementation Dublin 2020 [2].

^b^ New HIV diagnoses in 2020 among persons > 14 years with CD4^+^ T-cell count levels reported.

^c^ Completeness of number of cases with CD4^+^.

^d^ Data from HIV/AIDS surveillance in Europe 2020 [5].

Participating countries were European countries involved in the established infrastructure of the Optimising Testing and Linkage to Care for HIV across Europe (OptTEST) project.

Specific guidelines for the following eight HIV ICs were available in all countries: cervical cancer, cervical dysplasia, hepatitis C, malignant lymphoma/non-Hodgkin’s lymphoma, *Mycobacterium tuberculosis*, pregnancy, primary lung cancer, and sexually transmitted infections (STIs) (Table 2). Of these, the ICs pregnancy and STIs had at least one guideline available in all participating countries that recommended HIV testing. However, none of the 62 HIV ICs had HIV testing recommendations included in all their available guidelines in any participating country. The ICs cervical cancer, cervical dysplasia, and primary lung cancer had guidelines available that recommended HIV testing in two of the participating countries. None of the countries identified a disease-specific guideline for the ADC disseminated penicilliosis. The ADCs coccidioidomycosis, histoplasmosis, progressive multifocal leukoencephalopathy, reactivation of American trypanosomiasis and the non-AIDS-defining HIV IC unexplained fever were only covered by guidelines in one or two countries. Of these, the disease-specific guideline reactivation of American trypanosomiasis did not mention HIV, half of the available guidelines for the other three ADCs recommended HIV testing and both available guidelines for unexplained fever recommended HIV testing.

**Table 2:** Number of participating European countries with at least one national guideline available per HIV indicator condition and countries with missing HIV indicator condition guidelines, 2019–2021 (n = 15)

Speciality and HIV indicator condition	Number of countries with at least one guideline available	Number of countries with at least one guideline available that reports HIV association	Number of countries with at least one guideline available that recommends HIV testing	Countries missing the HIV IC guideline	Countries with guideline available for HIV IC without HIV test recommendation
**Dermatology / venereology**					
**Herpes simplex, ulcer(s) > I month/bronchitis/pneumonitis**	10	6	4	CH, DE, GR, LT, UK	BE, DK, NL, PL, RO, RU
**Kaposi's sarcoma**	10	9	4	BE, FR, GR, NL, RO	BY, DK, IT, LT, PL, UK
Herpes zoster	13	10	8	IT, LT	BE, GR, PL, RO, UK
Seborrhoeic dermatitis/exanthema	6	5	1	BE, CH, DE, FR, GR, IT, LT, PL, RO	BY, DK, NL, RU, UA
Severe or atypical psoriasis	14	10	8	BE	DK, FR, GR, LT, NL, UK
Sexually transmitted infections	15	15	15	None	None
**Gastroenterology / hepatology**					
**Candidiasis, oesophageal**	7	6	2	BY, CH, FR, IT, LT, PL, RO, RU	BE, DE, DK, GR, NL
**Cryptosporidiosis diarrhoea, > 1 month**	10	9	4	BE, CH, FR, RO, RU	DK, GR, LT, NL, PL, UK
**Cystoisosporiasis (formerly known as Isosporiasis) > 1 month**	8	6	3	CH, DE, DK, FR, LT, RO, RU	BE, GR, NL, PL, UK
Anal cancer/dysplasia	13	12	8	BE, RO	BY, GR, LT, RU, UA
Hepatitis A	10	3	2	BE, CH, IT, PL, UA	BY, DE, DK, FR, GR, LT, RO, UK
Hepatitis B (acute or chronic)	13	13	12	BE, CH	RU
Hepatitis C (acute or chronic)	15	15	13	None	BE, RU
Unexplained chronic diarrhoea	6	4	1	BE, CH, DE, DK, FR, IT, LT, PL, RU	BY, GR, NL, UA, UK
**Gynaecology / obstetrics**					
**Cervical cancer**	15	7	2	None	BE, CH, DE, DK, FR, GR, IT, LT, NL, PL, RO, RU, UK
Cervical dysplasia	15	8	2	None	BE, CH, DE, DK, FR, GR, IT, LT, NL, PL, RO, RU, UK
Pregnancy (implications for the unborn child)	15	15	15	None	None
**Haematology**					
**Non-Hodgkin lymphoma**	14	14	13	GR	RU
Castleman's disease	4	3	2	BE, CH, DE, FR, GR, IT, LT, NL, RO, UA, UK	BY, PL
Idiopathic/thrombotic thrombocytopenic purpura	14	10	9	LT	CH, DK, GR, PL, UA
Malignant lymphoma/Hodgkin's lymphoma	15	15	14	None	DK
**Internal medicine**					
**Atypical disseminated leishmaniasis**	8	6	3	BE, BY, CH, FR, GR, RO, RU	DK, IT, LT, NL, PL
**Candidiasis, bronchial/tracheal/lungs**	7	3	1	BY, CH, IT, LT, PL, RO, RU, UK	BE, DE, DK, F, GR, NL
**Coccidioidomycosis, disseminated/extrapulmonary**	2	2	1	BE, BY, CH, DE, FR, GR, IT, LT, NL, PL, RO, RU, UK	DK
**Cryptococcosis, extrapulmonary**	5	4	2	BY, CH, DE, FR, IT, LT, PL, RO, RU, UK	BE, GR, NL
**Cytomegalovirus, other (except liver, spleen, glands)**	6	6	2	CH, DE, DK, FR, GR, LT, RO, UA, UK	BE, NL, PL, RU
**Histoplasmosis, disseminated/extrapulmonary**	2	1	1	BE, BY, CH, DE, FR, GR, IT, LT, NL, PL, RO, RU, UK	DK
**Penicilliosis, disseminated**	0	0	0	BE, BY, CH, DE, DK, FR, GR, IT, LT, NL, PL, RO, RU, UA, UK	None
**Reactivation of American trypanosomiasis (meningoencephalitis or myocarditis)**	2	0	0	BE, BY, CH, DE, FR, GR, LT, NL, PL, RO, RU, UA, UK	DK, IT
***Salmonella* septicaemia, recurrent**	8	5	2	BY, CH, FR, IT, PL, RO, UK	BE, DE, DK, GR, LT, NL
Candidaemia	4	1	0	BE, BY, CH, DK, FR, IT, LT, RO, RU, UA, UK	DE, GR, NL, PL
Candidiasis	8	4	1	CH, DE, DK, FR, LT, PL, UK	BE, GR, IT, NL, RO, RU, UA
Invasive pneumococcal disease	5	3	0	BY, CH, DE, FR, GR, IT, PL, RO, RU, UA	BE, DK, LT, NL, UK
Mononucleosis-like illness	6	6	4	BE, CH, DE, FR, GR, IT, LT, PL, RO	DK, NL
Oral hairy leukoplakia	7	4	3	BE, CH, FR, GR, LT, NL, PL, RO	DE, DK, IT, UK
Unexplained chronic renal impairment	13	5	5	CH, DK	BE, DE, GR, LT, NL, PL, RU, UK
Unexplained fever	2	2	2	BE, BY, CH, DE, FR, GR, IT, LT, NL, PL, RO, RU, UA	None
Unexplained leukocytopenia/thrombocytopenia lasting > 4 weeks	8	7	6	BE, BY, GR, IT, LT, RO, RU	DK, PL
Unexplained lymphadenopathy	7	6	6	BE, BY, CH, FR, GR, LT, NL, RO	PL
Unexplained oral candidiasis	9	4	3	BY, CH, DK, F, GR, LT	BE, DE, IT, NL, PL, UA
Unexplained weight loss	4	2	1	BE, BY, CH, FR, GR, IT, LT, NL, PL, RU, UA	DE, DK, UK
Visceral leishmaniasis	6	5	2	BE, BY, CH, DK, FR, IT, PL, RU, UK	GR, LT, NL, RO
**Neurology / neurosurgery**					
**Cerebral toxoplasmosis**	8	8	3	CH, DE, FR, IT, RO, RU, UK	DK, GR, LT, NL, PL
**Primary cerebral lymphoma**	11	11	10	CH, GR, NL, RO	BY
**Progressive multifocal leukoencephalopathy**	2	1	1	BE, BY, CH, DK, FR, GR, IT, LT, NL, PL, RO, RU, UK	DE
Cerebral abscess	8	4	4	DK, FR, IT, LT, NL, RO, UK	GR, PL, RU, UA
Guillain–Barré́ syndrome	5	5	4	BE, CH, FR, GR, IT, LT, PL, RO, UA, UK	NL
Lymphocytic meningitis	9	7	4	CH, IT, LT, PL, RO, RU	BE, BY, DK, NL, UA
Mononeuritis	7	5	4	BE, CH, DE, LT, PL, RO, RU, UA	FR, GR, UK
Multiple sclerosis-like disease	8	5	3	BE, CH, FR, IT, PL, RU, UA	BY, DK, GR, LT, NL
Peripheral neuropathy	9	6	2	BE, CH, LT, PL, RU, UA	BY, DE, FR, GR, IT, NL, UK
Primary space occupying lesion of the brain	3	2	2	BE, CH, DE, DK, F, IT, LT, NL, PL, RO, UA, UK	RU
Subcortical dementia	10	9	6	BY, FR, LT, PL, RU	BE, NL, UA, UK
**Ophthalmology**					
**Cytomegalovirus retinitis**	7	7	3	BE, CH, FR, GR, IT, LT, RO, UK	DK, NL, PL, RU
Infective retinal diseases, including herpes viruses and toxoplasma	5	2	1	BE, CH, DE, DK, FR, GR, IT, NL, RO, RU	LT, PL, UA, UK
**Pulmonology**					
***Mycobacterium avium* complex or *Mycobacterium kansasii*, disseminated or extrapulmonary**	7	6	4	BE, CH, FR, GR, IT, LT, NL, RU	DE, DK, PL
***Mycobacterium*, other species or unidentified species, disseminated or extrapulmonary**	3	2	2	BE, BY, CH, DE, DK, FR, GR, IT, LT, NL, RU, UA	PL
***Mycobacterium tuberculosis,* pulmonary or extrapulmonary**	15	14	12	None	PL, RU, UK
***Pneumocystis carinii* pneumonia**	9	7	4	CH, DE, FR, IT, PL, UK	BE, GR, LT, NL, RU
**Pneumonia, recurrent (2 or more episodes in 12 months)**	11	4	2	FR, IT, PL, RO	BE, BY, DE, DK, LT, NL, RU, UA, UK
Community-acquired pneumonia	14	5	2	RO	BE, BY, CH, FR, GE, IT, LT, NL, PL, RU, UA, UK
Primary lung cancer	15	4	2	None	BE, BY, CH, DE, DK, GR, IT, LT, NL, PL, RO, RU, UK

BE: Belgium, BY: Belarus; CH: Switzerland; DE: Germany; DK: Denmark; FR: France; GR: Greece; IT: Italy; LT: Lithuania; NL: the Netherlands; PL: Poland; RO: Romania; RU: Russia; UA: Ukraine; UK:  United Kingdom.

AIDS-defining conditions are written in bold.

Participating countries were European countries involved in the established infrastructure of the Optimising Testing and Linkage to Care for HIV across Europe (OptTEST) project.

**Figure 1:**
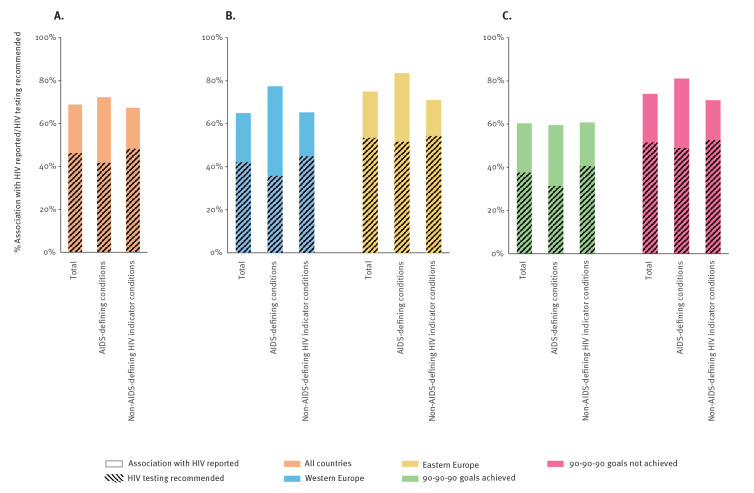
Proportions of identified HIV indicator condition guidelines that report the association with HIV (solid boxes) and recommend HIV testing (dashed boxes) for (A) all countries, (B) western Europe vs eastern Europe, (C) 90-90-90 goals achieved vs not achieved, 2019–2021 (n = 15)

Overall, the participating countries had at least one guideline available for 57% of the HIV ICs, including 56% and 58% for western (n = 9) and eastern (n = 6) European countries, respectively. Overall, 545 of the 791 (69%) identified guidelines reported the association with HIV and 366 of the 791 (46%) guidelines recommended HIV testing (The total number and proportions of identified HIV IC guidelines that report the association with HIV and recommend HIV testing overall, geographically ordered and according to achieved 90-90-90 goals can be found in Supplementary Table S3). Furthermore, 175/242 (72%) ADC guidelines and 370/549 (67%) non-AIDS-defining HIV IC guidelines reported an association with HIV, and 101/242 (42%) ADC guidelines and 265/549 (48%) non-AIDS-defining HIV IC guidelines recommended HIV testing (p = 0.089, Figure 1A). The recommendation to test was not associated with the year of guideline publication (p = 0.13) (The total number and proportions of identified HIV IC guidelines per publication year can be found in Supplementary Table S4). Using WHO guidelines as a reference, we found higher reported HIV association (overall 91%; 100% ADCs vs 88% non-AIDS-defining HIV ICs) and comparable testing recommendation rates (overall 50%; 56% ADCs vs 48% non-AIDS-defining HIV ICs) in the 34 guidelines covering 14 HIV ICs (4 ADCs and 10 non-AIDS-defining HIV ICs) (The total number and proportions of identified HIV IC guidelines in the WHO guideline database can be found in Supplementary Table S5).

The association with HIV was more frequently reported in HIV IC guidelines from eastern European countries and those countries yet to achieve the 90–90–90 goals (Figure 1B and 1C). The 288 guidelines identified in eastern European countries showed overall higher test recommendations than the 503 guidelines identified in western European countries (53% vs 42%, p = 0.002). This was mostly driven by the more frequent HIV testing recommendations in available ADC guidelines. A higher HIV testing recommendation uptake in guidelines was also observed in the countries that did not achieve the 90–90–90 goals than those that did achieve these goals (52% of 493 guidelines vs 38% of 298 guidelines, p < 0.001).

There was no HIV IC that had HIV testing universally recommended in all identified guidelines and had at least one guideline available in all participating countries (The total number and proportions of HIV IC guidelines that report HIV and recommend HIV testing per HIV IC can be found in Supplementary Table S6). When further evaluating the guidelines that were available in these countries, we found that the disease-specific guidelines for coccidioidomycosis, mononucleosis-like illness, non-Hodgkin lymphoma, and unexplained fever had a 100% coverage of mentioning the relationship with HIV. However, substantial differences were present in the total number of available guidelines per HIV IC, ranging from 0 to 76. Of the 62 HIV ICs, only one HIV IC (1.6%), unexplained fever, had 100% coverage of HIV testing recommendations, and four HIV ICs (6.5%), non-Hodgkin lymphoma, pregnancy, primary cerebral lymphoma and unexplained leukocytopenia/thrombocytopenia had more than 75% coverage of testing recommendations among the available guidelines. HIV testing recommendations were not included in any guidelines of American trypanosomiasis, candidaemia or invasive pneumococcal disease. In western Europe, the guidelines for candidiasis, candidaemia, cervical cancer, cervical dysplasia, oral hairy leukoplakia, *Salmonella* septicaemia, unexplained chronic diarrhoea and unexplained weight loss lacked any HIV testing recommendation, and in eastern Europe this applied to community-acquired pneumonia, recurrent pneumonia, and seborrhoeic dermatitis/exanthema (The total number and proportions of HIV IC guidelines that report HIV and recommend HIV testing per HIV IC geographically grouped can be found in Supplementary Table S7). In countries not yet achieving the 90–90–90 goals, 56 HIV ICs had at least one guideline with HIV testing recommendations available, although the median overall testing recommendation rates remained at 53% (The total number and proportions of HIV IC guidelines that report HIV and recommend HIV testing per HIV IC grouped according to achieved 90-90-90 goals can be found in Supplementary Table S8).

### HIV testing recommendation gaps in medical non-HIV specialty guidelines

**Figure 2:**
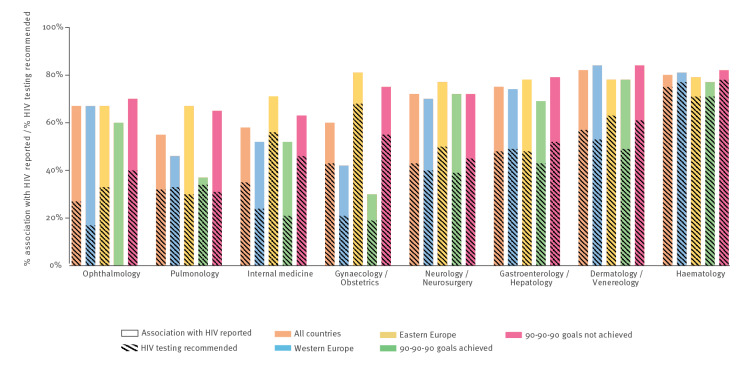
The proportions of identified HIV indicator condition guidelines that report the association with HIV and recommend HIV testing for all countries, for western and eastern European countries, and according to whether the 90–90–90 goals have been achieved, per specialty, 2019–2021 (n = 15)

Given the central role of medical specialty societies in national guideline development, we also analysed HIV testing recommendation uptake according to medical specialty (Figure 2) (The total number and proportions of HIV IC guidelines that report HIV and recommend HIV testing per HIV IC grouped according to achieved 90-90-90 goals can be found in Supplementary Table S9). Compared with the overall uptake of HIV testing recommendations in HIV IC guidelines (46%), guidelines for the specialties of dermatology/venereology, gastroenterology/hepatology, and haematology recommended HIV testing more often (range 48–75%). Guidelines on haematological conditions that at least reported the association with HIV (80%) also had the highest HIV testing recommendation rate (75%), whereas the average proportion of guidelines from all medical specialties that mentioned an association with HIV was 69%, with just 46% also recommending testing. The specialties gynaecology, internal medicine, neurology/neurosurgery, ophthalmology and pulmonology reported fewer overall HIV testing recommendation rates (range 27–43%). The largest discrepancies in the HIV testing recommendation uptake of guidelines between western and eastern European countries, and between countries with and without achieved 90–90–90 goals, were found in the specialties gynaecology (21% vs 68% and 19% vs 55%) and internal medicine (24% vs 56% and 21% vs 46%). Ophthalmology and pulmonology had the poorest reporting of HIV testing recommendations both overall (27% and 32%, respectively) and across all variables (western/eastern European countries and 90-90-90 goals achieved or not) (range 0–40% and 30–34%, respectively).

### HIV indicator condition guideline coverage cascade

**Figure 3:**
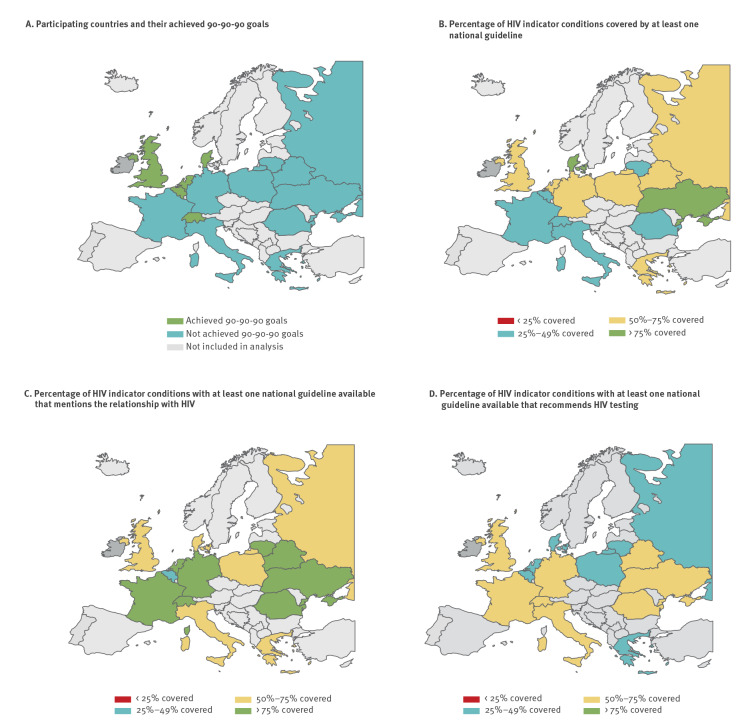
HIV indicator condition guideline coverage cascades for countries (A) that have achieved or not achieved the 90–90–90 goals, (B) the proportion of HIV indicator condition covered by at least one national guideline, (C) the proportion of the available guidelines with at least one guideline available that mentions the relationship with HIV, (D) the proportion of the available guidelines with at least one guideline available that recommends HIV testing, 2019–2021 (n = 15)

We analysed the HIV guideline coverage cascade in all participating countries and related it to the achievement of the 90–90–90 goals as of December 2021 (Figure 3A). The first pillar of the HIV IC guideline covering cascade (HIV IC guideline availability) was lowest in Switzerland (31%) and France (39%), and highest in Ukraine (76%) and Denmark (77%) (Figure 3B). The second pillar which represented mentioning the relationship with HIV where a guideline was available was lowest in Belgium (47%), and > 75% in Belarus, France, Germany, Lithuania, the Netherlands, Romania, Switzerland, and Ukraine (range 79–98%) (Figure 3C). Relevant gaps in the uptake of HIV testing recommendations where a guideline was available (third pillar) were identified in all countries (Figure 3D). The median coverage of the third pillar was 50% (IQR: 31–68%) with seven countries having HIV testing recommendations included in less than 50% of their available HIV IC guidelines (range 29–46%). The coverage of the third pillar was higher in eastern European countries than in western European countries (55% vs 46%) and in countries that had not yet achieved the 90–90–90 goals (54%) vs countries that had reached these goals (41%).

## Discussion

This European guideline review demonstrates that fewer than half of the national guidelines for HIV ICs recommend HIV testing. This observation was consistent across a wide range of HIV ICs, countries and medical specialties. Importantly, many guidelines for those HIV ICs known to be ADCs lacked HIV testing recommendations. These findings highlight clinically relevant gaps throughout Europe in the representation of HIV testing recommendations in disease-specific guidelines for HIV ICs. Since clinical practice guidelines represent a cornerstone of clinical medicine, ensuring guidelines for HIV ICs with a universal uptake of HIV testing recommendations should improve good clinical practice for people with undiagnosed HIV as it has in other fields of HIV medicine [14-16,27]. Ultimately, a timely HIV diagnosis can expedite treatment initiation which prevents disease progression, death and onward HIV transmission [28,29].

We did find some promising signals in our data. Firstly, all countries that did not yet achieve the 90-90-90 goals, and all eastern European countries regardless of whether they had achieved the 90-90-90 goals or not, had a higher uptake of HIV testing recommendations in their guidelines. Accurate HIV testing recommendations in guidelines can be regarded as the first step to better implementation of HIV testing in daily practice. The uptake of HIV testing recommendations in guidelines may be associated with improved identification of PLWH, as illustrated by the decreasing number of those undiagnosed in eastern Europe, although other factors likely determine the accurate testing in clinical practice, including available resources and stigma to test in these settings. Secondly, regarding medical specialties, haematology guidelines had a superior HIV testing recommendation uptake in available guidelines of related HIV ICs, with a homogenous pattern across Europe. Thirdly, two prevalent HIV ICs (pregnancy and STIs) had guidelines available in all European countries with at least one guideline recommending HIV testing in every country. This indicates that a high assimilation of HIV testing recommendations in guidelines across the European continent is possible.

Our study adds evidence to the current knowledge on the uptake of HIV testing recommendations in HIV IC guidelines. Studies conducted in the UK, Greece and Australia found HIV testing recommendations in 26% to 38% of the guidelines [20,30,31]. We introduce the concept of HIV IC guideline coverage cascades which can help monitor the current uptake and future progress of HIV testing recommendations and allow comparisons between settings and across time. In addition, our study highlights a more general omission in incorporating medical guidance from the WHO and ECDC on HIV IC-guided testing into national non-HIV specialty guidelines. This may indicate a possible lack of HIV expertise within these medical specialties’ guideline panels. Although medical specialties responsible for HIV care differ throughout Europe, no specialty comes close to a universal uptake of HIV testing recommendations. In order to begin to address this, we suggest consideration should be given to involving HIV medical specialists in relevant guideline development, better education of healthcare professionals on HIV IC-guided testing and support from national HIV patient associations. Where national HIV testing guidelines exist (or the country has a policy to follow ECDC or WHO guidelines), guideline authors and policymakers should be made aware of, and called on, to correct the lack of consistency across guidelines and policies. Informing and enabling the public to test for HIV upon indication and ensuring destigmatised HIV testing options for everyone remain important components which, if insufficiently ensured, can considerably hinder translation of guidelines into clinical practice and linkage to care [32-34].

This study has a number of potential limitations. Firstly, only half of the 30 countries approached provided data for this analysis. However, the countries included are the main European countries of the continental epidemic, which together cover ca 83% of the undiagnosed PLWH in Europe. Also, the cultures and 90–90–90 goals of the included countries are comparable to the countries that were approached, but did not provide any data for this review, making generalisability likely. Secondly, we acknowledge that subjectivity in data collection may have biased our findings. Therefore, several measures were taken to mitigate this. We used a standard operating procedure for the search strategy that was previously validated in one country. The data collection was focused on three simple key variables to limit the risk of individual interpretation. Thirdly, the level of the reviewers’ HIV expertise varied and the possibility to search for guidelines by online search engines could have introduced reporting bias. A pilot we ran in one country indicated that the same procedural instructions resulted in a high inter-observer guideline review interpretation agreement. This implies that there is no large systematic error in the HIV IC guideline coverage cascades. Fourthly, we cannot exclude the possibility that guidelines have been missed. However, the reviewers were recruited because of their HIV expertise and multiple reviewers were generally available per country, which reduced the risk of missing relevant guidelines. Finally, we focused on the availability of official national guidelines only. By not taking the potential recommended use of international guidelines for HIV ICs into account by physicians, we risk underestimating the actual availability and use of HIV IC-specific guidelines in practice in certain specialities in some countries. However, we felt that the presence of national guidelines in native languages and including country-specific information was optimal for supporting setting-specific HIV IC-guided testing.

### Conclusion

Within the European continent, HIV IC guidelines consistently missed opportunities to recommend HIV testing, contributing to the high levels of undiagnosed HIV and late HIV presentations observed across Europe. Given their influence on clinical practice, inclusion of HIV testing recommendations should improve HIV awareness and HIV testing practices among physicians, allowing earlier linkage to care and leading to reductions in late presentations and transmission.

## Supplementary Data

228000 Supplementary Material 1

## Supplementary Data

83000 Supplementary Material 2

## Supplementary Data

17000 Supplementary Material 3

## Supplementary Data

438000 Supplementary Tables
