# Supplementary material for: National medical specialty guidelines of HIV indicator conditions in Europe lack adequate HIV testing recommendations: a systematic guideline review
Source: Euro Surveill. 2022 Dec 1;27(48):2200338. doi: 10.2807/1560-7917.ES.2022.27.48.2200338 (PMC9716648; doi:10.2807/1560-7917.ES.2022.27.48.2200338)
Supplement: Supplementary Material 1 [file 22-00338_JORDANS_Supplement_S1.pdf]

This supplementary material is hosted by Eurosurveillance as supporting information alongside the article ' National medical specialty guidelines of HIV indicator conditions in Europe lack adequate HIV testing recommendations: a systematic guideline review', on behalf of the authors, who remain responsible for the accuracy and appropriateness of the content. The same standards for ethics, copyright, attributions and permissions as for the article apply. Supplements are not edited by Eurosurveillance and the journal is not responsible for the maintenance of any links or email addresses provided therein.

HIV Indicator Condition Guidelines Review

A quick guide on how to start
May 2018

1. **Background**

Provider initiated testing is important to achieve early diagnosis for people living with HIV. HIV indicator conditions (IC) are conditions associated with a higher HIV prevalence and where an HIV test should always be offered to the patient. However, clinicians managing ICs may be unaware of recommendations produced by HIV societies or the prevalence of undiagnosed HIV infection among the patients they see and which present with HIV indicator conditions.

The objective of this work is to review national medical non-HIV specialty guidelines on AIDS defining conditions (ADC) and HIV indicator conditions (IC) – and ascertain if HIV is discussed and testing recommended in the guidelines.

AIDS defining conditions (ADC) and HIV indicator conditions (IC)

- TB
- STI
- Hep B
- Hep C
- Herpes zoster
- Mononucleosis-like illness
- Pneumonia
- Seborrheic dermatitis
- Unexplained fever
- Pneumonia (CAP)
- Malignant lymphoma
- Anal dysplasia/ cancer
- Cervical dysplasia/cancer
- Unexplained leuko-/ thrombocytopenia

Please find an expanded list of ADC and IC here.

1. Step-by-step guide to Indicator Condition Guideline Review

a. Search for the national guidelines for each ADC and IC through relevant medical specialty societies and associations websites, national guideline body/National Board of Health websites, PubMed, google, etc.

Bear in mind not all conditions have guidelines, i.e. infectious mononucleosis-like symptoms, in that case, look for i.e. glandular fever, rubella etc.

b. Insert all identified guidelines into excel tables to create an overview – one for ICs and one for ADCs (example below for ICs):

	A	B	C	D	E	F	G	H	I	J
1	Indicator condition	Guideline reference	Link to guideline	Issued by: (eg specialty society)	Year last updated	Does guideline mention HIV testing (Y/N)?	Does guideline conform with HIV testing guidelines (Y/N)?	Quote(s) from guideline re HIV testing	Paragraph/section number(s) for quote(s)	Reviewed by (initials)
2	HIV indicator condition									
3	Respiratory									
4	Community-acquired pneumonia									
5	Invasive pneumococcal disease									
6	Aspergillos									
7	Neurology									
8	Guillan-Barré									
9										
10										
11						Dermatology				
12	Herpes zoster									
13										
14										

II. Third step is the analysis of identified guidelines:

Categorise identified guidelines into 3 groups:

a. HIV is *not* mentioned in the guideline
b. Association with HIV is mentioned but testing not recommended
c. Association with HIV is mentioned and testing is recommended

III. Describe the findings/results in a brief report or manuscript.

For further information on the process, please see the results from UK, Spain and Estonia in this publication by Dr Emily Lord, Stockdale AJ, Maleck R et al Evaluation of HIV testing recommendations in specialty guidelines for the management of HIV indicator conditions and watch the video presentation from Dr Emily Lord here

If you have any questions, please do not hesitate to contact [name and email redacted]
