# Supplementary material for: National medical specialty guidelines of HIV indicator conditions in Europe lack adequate HIV testing recommendations: a systematic guideline review
Source: Euro Surveill. 2022 Dec 1;27(48):2200338. doi: 10.2807/1560-7917.ES.2022.27.48.2200338 (PMC9716648; doi:10.2807/1560-7917.ES.2022.27.48.2200338)
Supplement: Supplementary Tables [file 22-00338_JORDANS_Supplementary_tables.pdf]

# Supplementary Tables

This supplementary material is hosted by *Eurosurveillance* as supporting information alongside the article ***'National medical specialty guidelines of HIV indicator conditions in Europe lack adequate HIV testing recommendations: a systematic guideline review'***, on behalf of the authors, who remain responsible for the accuracy and appropriateness of the content. The same standards for ethics, copyright, attributions and permissions as for the article apply. Supplements are not edited by *Eurosurveillance* and the journal is not responsible for the maintenance of any links or email addresses provided therein.

**Supplementary Table S1: List of all AIDS-defining conditions and non-AIDS-defining HIV indicator conditions.**

| **AIDS-defining conditions** | **Non-AIDS-defining HIV indicator conditions** |
|---|---|
| *Atypical disseminated leishmaniasis* | Anal cancer/dysplasia |
| *Candidiasis, bronchial/tracheal/lungs* | Candidaemia |
| *Candidiasis, oesophageal* | Candidiasis |
| *Cerebral toxoplasmosis* | Castleman's disease |
| *Cervical cancer* | Cerebral abscess |
| *Coccidioidomycosis, disseminated/extrapulmonary* | Cervical dysplasia |
| *Cryptococcosis, extrapulmonary* | Community-acquired pneumonia |
| *Cryptosporidiosis diarrhoea, >1 month* | Guillain–Barré syndrome |
| *Cytomegalovirus retinitis* | Hepatitis A |
| *Cytomegalovirus, other (except liver, spleen, glands)* | Hepatitis B (acute or chronic) |
| *Herpes simplex, ulcer(s) >I month/bronchitis/pneumonitis* | Hepatitis C (acute or chronic) |
| *Histoplasmosis, disseminated/extrapulmonary* | Herpes zoster |
| *Isosporiasis, >1 month* | Idiopathic/thrombotic thrombocytopenic purpura |
| *Kaposi's sarcoma* | Infective retinal diseases, including herpes viruses and toxoplasma |
| *Mycobacterium avium complex or Mycobacterium kansasii, disseminated or extrapulmonary* | Invasive pneumococcal disease |
| *Mycobacterium tuberculosis, pulmonary or extrapulmonary* | Lymphocytic meningitis |
| *Mycobacterium, other species or unidentified species, disseminated or extrapulmonary* | Malignant lymphoma/Hodgkin's lymphoma |
| *Non-Hodgkin lymphoma* | Mononeuritis |
| *Penicilliosis, disseminated* | Mononucleosis-like illness |
| *Pneumocystis carinii pneumonia* | Multiple sclerosis-like disease |
| *Pneumonia, recurrent (2 or more episodes in 12 months)* | Oral hairy leukoplakia |
| *Primary cerebral lymphoma* | Peripheral neuropathy |
| *Progressive multifocal leucoencephalopathy* | Pregnancy (implications for the unborn child) |
| *Reactivation of American trypanosomiasis (meningoencephalitis or myocarditis)* | Primary lung cancer |

| | |
|---|---|
| *Salmonella septicaemia, recurrent* | Primary space occupying lesion of the brain |
| | Seborrheic dermatitis/exanthema |
| | Severe or atypical psoriasis. |
| | Sexually transmitted infections |
| | Subcortical dementia |
| | Unexplained chronic diarrhoea |
| | Unexplained chronic renal impairment |
| | Unexplained fever |
| | Unexplained leukocytopenia/thrombocytopenia, >4 weeks |
| | Unexplained lymphadenopathy |
| | Unexplained oral candidiasis |
| | Unexplained weight loss |
| | Visceral leishmaniasis |

**Supplementary Table S2: All 62 HIV indicator conditions grouped per specialty.** AIDS-defining conditions are written in italics.

| | |
|---|---|
| **Dermatology / venereology** | *Herpes simplex, ulcer(s) >1 month/bronchitis/pneumonitis* |
| | *Kaposi's sarcoma* |
| | Herpes zoster |
| | Seborrheic dermatitis/exanthema |
| | Severe or atypical psoriasis |
| | Sexually transmitted infections |
| **Gastroenterology / hepatology** | *Candidiasis, oesophageal* |
| | *Cryptosporidiosis diarrhoea, >1 month* |
| | *Isosporiasis, >1 month* |
| | Anal cancer/dysplasia |
| | Hepatitis A |
| | Hepatitis B (acute or chronic) |
| | Hepatitis C (acute or chronic) |
| | Unexplained chronic diarrhoea |
| **Gynaecology / obstetrics** | *Cervical cancer* |
| | Cervical dysplasia |
| | Pregnancy (implications for the unborn child) |
| **Haematology** | *Non-Hodgkin lymphoma* |
| | Castleman's disease |
| | Idiopathic/thrombotic thrombocytopenic purpura |
| | Malignant lymphoma/Hodgkin's lymphoma |
| **Internal medicine** | *Atypical disseminated leishmaniasis* |
| | *Candidiasis, bronchial/tracheal/lungs* |
| | *Coccidioidomycosis, disseminated/extrapulmonary* |
| | *Cryptococcosis, extrapulmonary* |
| | *Cytomegalovirus, other (except liver, spleen, glands)* |
| | *Histoplasmosis, disseminated/extrapulmonary* |
| | *Penicilliosis, disseminated* |
| | *Reactivation of American trypanosomiasis (meningoencephalitis or myocarditis)* |
| | *Salmonella septicaemia, recurrent* |
| | Candidaemia |
| | Candidiasis |
| | Invasive pneumococcal disease |
| | Mononucleosis-like illness |
| | Oral hairy leukoplakia |
| | Unexplained chronic renal impairment |
| | Unexplained fever |

| | |
|---|---|
| | Unexplained leukocytopenia/thrombocytopenia lasting >4 weeks |
| | Unexplained lymphadenopathy |
| | Unexplained oral candidiasis |
| | Unexplained weight loss |
| | Visceral leishmaniasis |
| **Neurology / neurosurgery** | *Cerebral toxoplasmosis* |
| | *Primary cerebral lymphoma* |
| | *Progressive multifocal leucoencephalopathy* |
| | Cerebral abscess |
| | Guillain–Barré syndrome |
| | Lymphocytic meningitis |
| | Mononeuritis |
| | Multiple sclerosis-like disease |
| | Peripheral neuropathy |
| | Primary space occupying lesion of the brain |
| | Subcortical dementia |
| **Ophthalmology** | *Cytomegalovirus retinitis* |
| | Infective retinal diseases, including herpes viruses and toxoplasma |
| **Pulmonology** | *Mycobacterium avium complex or Mycobacterium kansasii, disseminated or extrapulmonary* |
| | *Mycobacterium, other species or unidentified species, disseminated or extrapulmonary* |
| | *Mycobacterium tuberculosis, pulmonary or extrapulmonary* |
| | *Pneumocystis carinii pneumonia* |
| | *Pneumonia, recurrent (2 or more episodes in 12 months)* |
| | Community-acquired pneumonia |
| | Primary lung cancer |

**Supplementary Table S3: Total number and proportions of identified HIV indicator condition guidelines that report the association with HIV and recommend HIV testing overall, geographically ordered (Western versus Eastern Europe) and according to achieved 90-90-90 goals (90-90-90 goals achieved versus not yet achieved) and grouped according to AIDS-defining conditions and non-AIDS-defining HIV indicator conditions**

| | Number of identified guidelines | Association with HIV reported n (%) | HIV testing recommended n (%) |
|---|---|---|---|
| **All Guidelines** | **791** | **545 (69)** | **366 (46)** |
| AIDS-defining conditions | 242 | 175 (72) | 101 (42) |
| Non-AIDS-defining HIV indicator conditions | 549 | 370 (67) | 265 (48) |
| **Western Europe** | **503** | **329 (65)** | **212 (42)** |
| AIDS-defining conditions | 151 | 117 (77) | 54 (36) |
| Non-AIDS-defining HIV indicator conditions | 352 | 230 (65) | 158 (45) |
| **Eastern Europe** | **288** | **216 (75)** | **154 (53)** |
| AIDS-defining conditions | 91 | 76 (84) | 47 (52) |
| Non-AIDS-defining HIV indicator conditions | 197 | 140 (71) | 107 (54) |
| **90-90-90 goals achieved** | **298** | **180 (60)** | **112 (38)** |
| AIDS-defining conditions | 99 | 59 (60) | 31 (31) |
| Non-AIDS-defining HIV indicator conditions | 199 | 121 (61) | 81 (41) |
| **90-90-90 goals not yet achieved** | **493** | **365 (74)** | **254 (52)** |
| AIDS-defining conditions | 143 | 116 (81) | 70 (49) |
| Non-AIDS-defining HIV indicator conditions | 350 | 249 (71) | 184 (53) |

HIV = human immunodeficiency virus, AIDS = acquired immune deficiency syndrome

**Supplementary Table S4: Total number and proportions of identified HIV indicator condition guidelines per publication year.**

| Publication year guideline | Total number of guidelines<br>n | Report HIV<br>n (%) | Recommend HIV testing<br>n (%) |
|---|---|---|---|
| 1997 | 0 | 0 | 0 |
| 1998 | 1 | 1<br>(100) | 1<br>(100) |
| 1999 | 2 | 2<br>(100) | 2<br>(100) |
| 2000 | 0 | 0 | 0 |
| 2001 | 3 | 2<br>(67) | 2<br>(67) |
| **Subtotal 1997 - 2001** | **6** | **5<br>(83)** | **5<br>(83)** |
| 2002 | 2 | 2<br>(100) | 1<br>(50) |
| 2003 | 23 | 20<br>(87) | 17<br>(74) |
| 2004 | 6 | 4<br>(67) | 3<br>(50) |
| 2005 | 6 | 1<br>(17) | 0 |
| 2006 | 6 | 4<br>(67) | 2<br>(33) |
| **Subtotal 2002 - 2006** | **43** | **31<br>(72)** | **23<br>(53)** |
| 2007 | 19 | 13<br>(68) | 8<br>(42) |
| 2008 | 24 | 19<br>(79) | 15<br>(63) |
| 2009 | 23 | 17<br>(74) | 13<br>(57) |
| 2010 | 36 | 26<br>(72) | 20<br>(56) |
| 2011 | 30 | 21<br>(70) | 13<br>(43) |
| **Subtotal 2007 - 2011** | **132** | **96<br>(73)** | **69<br>(52)** |

| | | | |
|---|---|---|---|
| 2012 | 43 | 17<br>(40) | 12<br>(28) |
| 2013 | 35 | 23<br>(66) | 14<br>(40) |
| 2014 | 59 | 45<br>(76) | 31<br>(53) |
| 2015 | 57 | 41<br>(72) | 25<br>(44) |
| 2016 | 102 | 79<br>(77) | 54<br>(53) |
| **Subtotal 2012 - 2016** | **296** | **205<br>(69)** | **136<br>(46)** |
| 2017 | 58 | 36<br>(62) | 22<br>(38) |
| 2018 | 117 | 81<br>(69) | 54<br>(46) |
| 2019 | 74 | 55<br>(74) | 35<br>(47) |
| 2020 | 29 | 19<br>(66) | 11<br>(38) |
| 2021 | 27 | 15<br>(56) | 10<br>(38) |
| **Subtotal 2017-2021** | **305** | **206<br>(68)** | **132<br>(43)** |
| Publication year unknown | 9 | 2<br>(22) | 1<br>(11) |

HIV = human immunodeficiency virus

**Supplementary Table S5: Total number and proportions of identified HIV indicator condition guidelines in the WHO guideline database grouped by specialty.** AIDS-defining conditions are written in italics.

| | **Total n** | **Report HIV n (%)** | **Recommend HIV testing n (%)** |
|---|---|---|---|
| **Dermatology / venereology** | | | |
| *Herpes simplex, ulcer(s) >1 month/bronchitis/pneumonitis* | 1 | 1 (100) | 0 |
| *Kaposi's sarcoma* | 0 | 0 | 0 |
| Herpes zoster | 1 | 1 (100) | 1 (100) |
| Seborrheic dermatitis/exanthema | 0 | 0 | 0 |
| Severe or atypical psoriasis | 0 | 0 | 0 |
| Sexually transmitted infections | 9 | 7 (78) | 3 (33) |
| **Gastroenterology / hepatology** | | | |
| *Candidiasis, oesophageal* | 0 | 0 | 0 |
| *Cryptosporidiosis diarrhoea, >1 month* | 0 | 0 | 0 |
| *Isosporiasis, >1 month* | 0 | 0 | 0 |
| Anal cancer/dysplasia | 2 | 2 (100) | 1 (50) |
| Hepatitis A | 0 | 0 | 0 |
| Hepatitis B (acute or chronic) | 2 | 2 (100) | 1 (50) |
| Hepatitis C (acute or chronic) | 4 | 4 (100) | 2 (50) |
| Unexplained chronic diarrhoea | 0 | 0 | 0 |
| **Gynaecology / obstetrics** | | | |
| *Cervical cancer* | 2 | 2 (100) | 1 (50) |
| Cervical dysplasia | 2 | 2 (100) | 1 (50) |
| Pregnancy (implications for the unborn child) | 2 | 2 (100) | 2 (100) |
| **Haematology** | | | |

| | | | |
|---|---|---|---|
| *Non-Hodgkin lymphoma* | 0 | 0 | 0 |
| Castleman's disease | 0 | 0 | 0 |
| Idiopathic/thrombotic thrombocytopenic purpura | 0 | 0 | 0 |
| Malignant lymphoma/Hodgkin's lymphoma | 0 | 0 | 0 |
| **Internal medicine** | | | |
| *Atypical disseminated leishmaniasis* | 0 | 0 | 0 |
| *Candidiasis, bronchial/tracheal/lungs* | 0 | 0 | 0 |
| *Coccidioidomycosis, disseminated/extrapulmonary* | 0 | 0 | 0 |
| *Cryptococcosis, extrapulmonary* | 1 | 1 (100) | 0 |
| *Cytomegalovirus, other (except liver, spleen, glands)* | 0 | 0 | 0 |
| *Histoplasmosis, disseminated/extrapulmonary* | 0 | 0 | 0 |
| *Penicilliosis, disseminated* | 0 | 0 | 0 |
| *Reactivation of American trypanosomiasis (meningoencephalitis or myocarditis)* | 0 | 0 | 0 |
| *Salmonella septicaemia, recurrent* | 0 | 0 | 0 |
| Candidaemia | 0 | 0 | 0 |
| Candidiasis | 0 | 0 | 0 |
| Invasive pneumococcal disease | 0 | 0 | 0 |
| Mononucleosis-like illness | 0 | 0 | 0 |
| Oral hairy leukoplakia | 0 | 0 | 0 |
| Unexplained chronic renal impairment | 0 | 0 | 0 |
| Unexplained fever | 1 | 1 (100) | 1 (100) |
| Unexplained leukocytopenia/thrombocytopenia lasting >4 weeks | 0 | 0 | 0 |
| Unexplained lymphadenopathy | 0 | 0 | 0 |
| Unexplained oral candidiasis | 0 | 0 | 0 |
| Unexplained weight loss | 0 | 0 | 0 |
| Visceral leishmaniasis | 0 | 0 | 0 |
| **Neurology / neurosurgery** | | | |
| *Cerebral toxoplasmosis* | 0 | 0 | 0 |
| *Primary cerebral lymphoma* | 0 | 0 | 0 |
| *Progressive multifocal leucoencephalopathy* | 0 | 0 | 0 |
| Cerebral abscess | 0 | 0 | 0 |
| Guillain–Barré syndrome | 0 | 0 | 0 |
| Lymphocytic meningitis | 1 | 0 | 0 |
| Mononeuritis | 0 | 0 | 0 |
| Multiple sclerosis-like disease | 0 | 0 | 0 |

| | | | |
|---|---|---|---|
| Peripheral neuropathy | 0 | 0 | 0 |
| Primary space occupying lesion of the brain | 0 | 0 | 0 |
| Subcortical dementia | 1 | 1<br>(100) | 0 |
| **Ophthalmology** | | | |
| *Cytomegalovirus retinitis* | 0 | 0 | 0 |
| Infective retinal diseases, including herpes viruses and toxoplasma | 0 | 0 | 0 |
| **Pulmonology** | | | |
| *Mycobacterium avium complex (MAC) or Mycobacterium kansasii, disseminated or extrapulmonary* | 0 | 0 | 0 |
| *Mycobacterium, other species or unidentified species, disseminated or extrapulmonary* | 0 | 0 | 0 |
| *Mycobacterium tuberculosis, pulmonary or extrapulmonary* | 5 | 5<br>(100) | 4<br>(80) |
| *Pneumocystis carinii pneumonia* | 0 | 0 | 0 |
| *Pneumonia, recurrent (2 or more episodes in 12 months)* | 0 | 0 | 0 |
| Community-acquired pneumonia | 0 | 0 | 0 |
| Primary lung cancer | 0 | 0 | 0 |
| | | | |
| **Total** | **34** | **31<br>(91)** | **17<br>(50)** |
| AIDS-defining conditions | 9 | 9<br>(100) | 5<br>(56) |
| Non-AIDS-defining HIV indicator conditions | 25 | 22<br>(88) | 12<br>(48) |

HIV = human immunodeficiency virus, AIDS = acquired immune deficiency syndrome

**Supplementary Table S6: Total number and proportions of HIV indicator condition guidelines that report HIV and recommend HIV testing per HIV indicator condition.** AIDS-defining conditions are written in italics.

| | **Total n** | **Report HIV n (%)** | **Recommend HIV testing n (%)** |
|---|---|---|---|
| **Dermatology / venereology** | | | |
| *Herpes simplex, ulcer(s) >1 month/bronchitis/pneumonitis* | 12 | 8 (67) | 5 (42) |
| *Kaposi's sarcoma* | 11 | 9 (82) | 4 (36) |
| Herpes zoster | 16 | 12 (75) | 10 63) |
| Seborrheic dermatitis/exanthema | 7 | 5 (71) | 1 (14) |
| Severe or atypical psoriasis | 20 | 11 (55) | 8 (40) |
| Sexually transmitted infections | 76 | 71 (93) | 53 (70) |
| **Gastroenterology / hepatology** | | | |
| *Candidiasis, oesophageal* | 7 | 6 (86) | 2 (29) |
| *Cryptosporidiosis diarrhoea, >1 month* | 13 | 11 (85) | 4 (31) |
| *Isosporiasis, >1 month* | 9 | 6 (67) | 3 (33) |
| Anal cancer/dysplasia | 18 | 16 (89) | 11 (61) |
| Hepatitis A | 13 | 3 (23) | 2 (15) |
| Hepatitis B (acute or chronic) | 28 | 24 (86) | 19 (68) |
| Hepatitis C (acute or chronic) | 25 | 20 (80) | 16 (64) |
| Unexplained chronic diarrhea | 7 | 4 (57) | 1 (14) |
| **Gynaecology / obstetrics** | | | |

| | | | |
|---|---|---|---|
| *Cervical cancer* | 25 | 8<br>(32) | 2<br>(8) |
| Cervical dysplasia | 24 | 11<br>(46) | 3<br>(13) |
| Pregnancy (implications for the unborn child) | 31 | 29<br>(94) | 29<br>(94) |
| **Haematology** | | | |
| *Non-Hodgkin lymphoma* | 22 | 22<br>(100) | 21<br>(95) |
| Castleman's disease | 4 | 3<br>(75) | 2<br>(50) |
| Idiopathic/thrombotic thrombocytopenic purpura | 19 | 15<br>(79) | 14<br>(74) |
| Malignant lymphoma/Hodgkin's lymphoma | 36 | 25<br>(69) | 24<br>(67) |
| **Internal medicine** | | | |
| *Atypical disseminated leishmaniasis* | 11 | 8<br>(73) | 4<br>(36) |
| *Candidiasis, bronchial/tracheal/lungs* | 7 | 3<br>(43) | 1<br>(14) |
| *Coccidioidomycosis, disseminated/extrapulmonary* | 2 | 2<br>(100) | 1<br>(50) |
| *Cryptococcosis, extrapulmonary* | 5 | 4<br>(80) | 2<br>(40) |
| *Cytomegalovirus, other (except liver, spleen, glands)* | 8 | 6<br>(75) | 2<br>(25) |
| *Histoplasmosis, disseminated/extrapulmonary* | 2 | 1<br>(50) | 1<br>(50) |
| *Penicilliosis, disseminated* | 0 | 0 | 0 |
| *Reactivation of American trypanosomiasis (meningoencephalitis or myocarditis)* | 1 | 0 | 0 |
| *Salmonella septicaemia, recurrent* | 9 | 5<br>(56) | 2<br>(22) |
| Candidaemia | 5 | 1<br>(20) | 0 |
| Candidiasis | 11 | 5<br>(45) | 1<br>(9) |

| | | | |
|---|---|---|---|
| Invasive pneumococcal disease | 7 | 3 (43) | 0 |
| Mononucleosis-like illness | 8 | 8 (100) | 5 (63) |
| Oral hairy leukoplakia | 8 | 4 (50) | 3 (38) |
| Unexplained chronic renal impairment | 19 | 5 (26) | 5 (26) |
| Unexplained fever | 3 | 3 (100) | 3 (100) |
| Unexplained leukocytopenia/thrombocytopenia lasting >4 weeks | 10 | 9 (90) | 8 (80) |
| Unexplained lymphadenopathy | 9 | 6 (67) | 6 (67) |
| Unexplained oral candidiasis | 8 | 4 (50) | 3 (38) |
| Unexplained weight loss | 4 | 2 (50) | 1 (25) |
| Visceral leishmaniasis | 6 | 5 (83) | 2 (33) |
| **Neurology / neurosurgery** | | | |
| *Cerebral toxoplasmosis* | 9 | 8 (89) | 3 (33) |
| *Primary cerebral lymphoma* | 13 | 12 (92) | 10 (77) |
| *Progressive multifocal leucoencephalopathy* | 2 | 1 (50) | 1 (50) |
| Cerebral abscess | 9 | 5 (56) | 4 (44) |
| Guillain–Barré syndrome | 7 | 5 (71) | 4 (57) |
| Lymphocytic meningitis | 10 | 7 (70) | 4 (40) |
| Mononeuritis | 7 | 5 (71) | 4 (57) |
| Multiple sclerosis-like disease | 14 | 8 (57) | 3 (21) |
| Peripheral neuropathy | 12 | 9 (75) | 2 (17) |

| | | | |
|---|---|---|---|
| Primary space occupying lesion of the brain | 3 | 2<br>(67) | 2<br>(67) |
| Subcortical dementia | 14 | 10<br>(71) | 6<br>(43) |
| **Ophthalmology** | | | |
| *Cytomegalovirus retinitis* | 9 | 8<br>(89) | 3<br>(33) |
| Infective retinal diseases including herpes viruses and toxoplasma | 6 | 2<br>(33) | 1<br>(17) |
| **Pulmonology** | | | |
| *Mycobacterium avium complex (MAC) or Mycobacterium kansasii, disseminated or extrapulmonary* | 7 | 6<br>(86) | 4<br>(57) |
| *Mycobacterium, other species or unidentified species, disseminated or extrapulmonary* | 3 | 2<br>(67) | 2<br>(67) |
| *Mycobacterium tuberculosis, pulmonary or extrapulmonary* | 31 | 27<br>(87) | 18<br>(58) |
| *Pneumocystis carinii pneumonia* | 9 | 7<br>(78) | 4<br>(44) |
| *Pneumonia, recurrent* | 14 | 5<br>(36) | 2<br>(14) |
| Community-acquired pneumonia | 18 | 8<br>(44) | 2<br>(11) |
| Primary lung cancer | 27 | 5<br>(19) | 3<br>(11) |

HIV = human immunodeficiency virus

**Supplementary Table S7: The total number and proportions of HIV indicator condition guidelines that report HIV and recommend HIV testing per HIV indicator condition geographically grouped.** AIDS-defining conditions are written in italics.

| | **Western Europe** | | | **Eastern Europe** | | |
|---|---|---|---|---|---|---|
| | **Total** | **Report HIV n (%)** | **Recommend HIV testing n (%)** | **Total** | **Report HIV n (%)** | **Recommend HIV testing n (%)** |
| **Dermatology / venereology** | | | | | | |
| *Herpes simplex, ulcer(s) >1 month/bronchitis/pneumonitis* | 6 | 4 (67) | 2 (33) | 6 | 4 (67) | 3 (50) |
| *Kaposi's sarcoma* | 5 | 5 (100) | 2 (40) | 6 | 4 (67) | 2 (33) |
| Herpes zoster | 9 | 6 (67) | 5 (56) | 7 | 6 (86) | 5 (71) |
| Seborrheic dermatitis/exanthema | 3 | 3 (100) | 1 (33) | 4 | 2 (50) | 0 |
| Severe or atypical psoriasis | 13 | 6 (46) | 3 (23) | 7 | 5 (71) | 5 (71) |
| Sexually transmitted infections | 52 | 50 (96) | 34 (65) | 24 | 21 (88) | 19 (79) |
| **Subtotal** | **88** | **74 (84)** | **47 (53)** | **54** | **42 (78)** | **34 (63)** |
| **Gastroenterology / hepatology** | | | | | | |
| *Candidiasis, oesophageal* | 6 | 5 (83) | 1 (17) | 1 | 1 (100) | 1 (100) |
| *Cryptosporidiosis diarrhoea, >1 month* | 9 | 7 (78) | 2 (22) | 4 | 4 (100) | 2 (50) |
| *Isosporiasis, >1 month* | 6 | 4 (67) | 1 (17) | 3 | 2 (67) | 2 (67) |
| Anal cancer/dysplasia | 12 | 12 (100) | 10 (83) | 6 | 4 (67) | 1 (17) |
| Hepatitis A | 9 | 1 (11) | 1 (11) | 4 | 2 (50) | 1 (25) |
| Hepatitis B (acute or chronic) | 18 | 16 (89) | 13 (72) | 10 | 8 (80) | 6 (60) |
| Hepatitis C (acute or chronic) | 16 | 12 (75) | 11 (69) | 9 | 8 (89) | 5 (56) |

| | | | | | | |
|---|---|---|---|---|---|---|
| Unexplained chronic diarrhea | 4 | 2 (50) | 0 | 3 | 2 (67) | 1 (33) |
| **Subtotal** | **79** | **58 (73)** | **39 (49)** | **38** | **30 (79)** | **19 (50)** |
| **Gynaecology / obstetrics** | | | | | | |
| *Cervical cancer* | 16 | 3 (19) | 0 | 9 | 5 (56) | 2 (22) |
| Cervical dysplasia | 17 | 6 (35) | 0 | 7 | 5 (71) | 3 (43) |
| Pregnancy (implications for the unborn child) | 10 | 9 (90) | 9 (90) | 21 | 20 (95) | 20 (95) |
| **Subtotal** | **43** | **18 (42)** | **9 (21)** | **37** | **30 (81)** | **25 (68)** |
| **Haematology** | | | | | | |
| *Non-Hodgkin lymphoma* | 15 (100) | 15 (100) | 15 (100) | 9 | 5 (56) | 2 (22) |
| Castleman's disease | 1 | 1 (100) | 1 (100) | 3 | 2 (67) | 1 (33) |
| Idiopathic/Thrombotic thrombocytopenic purpura | 14 | 12 (86) | 11 (79) | 5 | 3 (60) | 3 (60) |
| Malignant lymphoma/Hodgkin's lymphoma | 27 | 18 (67) | 17 (63) | 9 | 7 (78) | 7 (78) |
| **Subtotal** | **57** | **46 (81)** | **44 (77)** | **24** | **19 (79)** | **17 (71)** |
| **Internal medicine** | | | | | | |
| *Atypical disseminated leishmaniasis* | 8 | 6 (75) | 3 (38) | 3 | 2 (67) | 1 (33) |
| *Candidiasis, bronchial/tracheal/lungs* | 6 | 2 (33) | 0 | 1 | 1 (100) | 1 (100) |
| *Coccidioidomycosis, disseminated/extrapulmonary* | 1 | 0 | 0 | 1 | 1 (100) | 1 (100) |
| *Cryptococcosis, extrapulmonary* | 4 | 3 (75) | 1 (25) | 1 | 1 (100) | 1 (100) |
| *Cytomegalovirus, other (except liver, spleen, glands)* | 5 | 3 (60) | 1 (20) | 4 | 4 (100) | 2 (50) |
| *Histoplasmosis, disseminated/extrapulmonary* | 1 | 0 | 0 | 1 | 1 (100) | 1 (100) |

| | | | | | | |
|---|---|---|---|---|---|---|
| *Penicilliosis, disseminated* | 0 | 0 | 0 | 0 | 0 | 0 |
| *Reactivation of American trypanosomiasis (meningoencephalitis or myocarditis)* | 2 | 0 | 0 | 0 | 0 | 0 |
| *Salmonella septicaemia, recurrent* | 6 | 2 (33) | 0 | 3 | 3 (100) | 2 (67) |
| Candidaemia | 4 | 1 (25) | 0 | 1 | 0 | 0 |
| Candidiasis | 6 | 3 (50) | 0 | 5 | 2 (40) | 1 (20) |
| Invasive pneumococcal disease | 6 | 2 (33) | 0 | 1 | 1 (100) | 0 |
| Mononucleosis-like illness | 4 | 4 (100) | 1 (25) | 4 | 4 (100) | 4 (100) |
| Oral hairy leukoplakia | 5 | 1 (20) | 0 | 3 | 3 (100) | 3 (100) |
| Unexplained chronic renal impairment | 11 | 2 (18) | 2 (18) | 8 | 3 (38) | 3 (38) |
| Unexplained fever | 2 | 2 (100) | 2 (100) | 1 | 1 (100) | 1 (100) |
| Unexplained leukocytopenia/thrombocytopenia lasting >4 weeks | 8 | 8 (100) | 7 (88) | 2 | 1 (50) | 1 (50) |
| Unexplained lymphadenopathy | 6 | 4 (67) | 4 (67) | 3 | 2 (67) | 2 (67) |
| Unexplained oral candidiasis | 5 | 2 (40) | 1 (20) | 3 | 2 (67) | 2 (67) |
| Unexplained weight loss | 3 | 1 (33) | 0 | 1 | 1 (100) | 1 (100) |
| Visceral leishmaniasis | 3 | 3 (100) | 1 (33) | 3 | 2 (67) | 1 (33) |
| **subtotal** | **95** | **50 (53)** | **23 (24)** | **48** | **34 (71)** | **27 (56)** |
| **Neurology / neurosurgery** | | | | | | |
| *Cerebral toxoplasmosis* | 5 | 4 (80) | 1 (20) | 4 | 4 (100) | 2 (50) |
| *Primary cerebral lymphoma* | 8 | 7 (88) | 6 (75) | 5 | 5 (100) | 4 (80) |
| *Progressive multifocal leukoencephalopathy* | 1 | 0 | 0 | 1 | 1 (100) | 1 (100) |

| | | | | | | |
|---|---|---|---|---|---|---|
| Cerebral abscess | 4 | 3 (75) | 3 (75) | 5 | 2 (40) | 1 (20) |
| Guillain–Barré syndrome | 5 | 3 (60) | 2 (40) | 2 | 2 (100) | 2 (100) |
| Lymphocytic meningitis | 7 | 6 (86) | 4 (57) | 3 | 1 (33) | 0 |
| Mononeuritis | 6 | 4 (67) | 3 (50) | 1 | 1 (100) | 1 (100) |
| Multiple sclerosis-like disease | 11 | 6 (55) | 2 (18) | 3 | 2 (67) | 1 (33) |
| Peripheral neuropathy | 1 | 1 (100) | 1 (100) | 2 | 1 (50) | 1 (50) |
| Primary space occupying lesion of the brain | 10 | 7 (70) | 1 (10) | 2 | 2 (100) | 1 (50) |
| Subcortical dementia | 12 | 8 (67) | 5 (42) | 2 | 2 (100) | 1 (50) |
| **Subtotal** | **70** | **49 (70)** | **28 (40)** | **30** | **23 (77)** | **15 (50)** |
| **Ophtalmology** | | | | | | |
| *Cytomegalovirus retinitis* | 5 | 4 (80) | 1 (20) | 4 | 4 (100) | 2 (50) |
| Infective retinal diseases, including herpes viruses and toxoplasma | 1 | 0 | 0 | 5 | 2 (40) | 1 (20) |
| **Subtotal** | **6** | **4 (67)** | **1 (17)** | **9** | **6 (67)** | **3 (33)** |
| **Pulmonology** | | | | | | |
| *Mycobacterium avium complex or Mycobacterium kansasii, disseminated or extrapulmonary* | 3 | 2 (67) | 1 (33) | 4 | 4 (100) | 3 (75) |
| *Mycobacterium, other species or unidentified species, disseminated or extrapulmonary* | 1 | 1 (100) | 1 (100) | 2 | 1 (50) | 1 (50) |
| *Mycobacterium tuberculosis, pulmonary or extrapulmonary* | 19 | 16 (84) | 13 (68) | 12 | 11 (92) | 5 (42) |
| *Pneumocystis carinii pneumonia* | 4 | 2 (50) | 1 (25) | 5 | 5 (100) | 3 (60) |
| *Pneumonia, recurrent (2 or more episodes in 12 months)* | 9 | 3 (33) | 2 (22) | 5 | 2 (40) | 0 |
| Community-acquired pneumonia | 10 | 4 | 2 | 8 | 4 | 0 |

| | | | | | | |
|---|---|---|---|---|---|---|
| | | (40) | (20) | | (50) | |
| Primary lung cancer | 17 | 1<br>(6) | 1<br>(6) | 10 | 4<br>(40) | 2<br>(20) |
| **Subtotal** | **63** | **29<br>(46)** | **21<br>(33)** | **46** | **31<br>(67)** | **14<br>(30)** |

HIV = human immunodeficiency virus

**Supplementary Table S8: Total number and proportions of HIV indicator condition guidelines that report HIV and recommend HIV testing per HIV indicator condition grouped according to achieved 90-90-90 goals.** AIDS-defining conditions are written in italics.

| | 90 – 90 – 90 goals achieved | | | 90 – 90 – 90 goals not achieved | | |
|---|---|---|---|---|---|---|
| | **Total** | **Report HIV n (%)** | **Recommend HIV testing n (%)** | **Total** | **Report HIV n (%)** | **Recommend HIV testing n (%)** |
| **Dermatology / venereology** | | | | | | |
| *Herpes simplex, ulcer(s) >1 month/bronchitis/pneumonitis* | 4 | 2 (50) | 0 | 8 | 6 (75) | 5 (63) |
| *Kaposi's sarcoma* | 3 | 3 (100) | 1 (33) | 8 | 6 (75) | 3 (38) |
| Herpes zoster | 6 | 3 (50) | 3 (50) | 10 | 9 (90) | 7 (70) |
| Seborrheic dermatitis/exanthema | 3 | 3 (100) | 1 (33) | 4 | 2 (50) | 0 |
| Severe or atypical psoriasis | 9 | 3 (33) | 1 (11) | 11 | 8 (73) | 7 (64) |
| Sexually transmitted infections | 24 | 24 (100) | 18 (75) | 52 | 47 (90) | 35 (67) |
| **Subtotal** | **49** | **38 (78)** | **24 (49)** | **93** | **78 (84)** | **57 (61)** |
| **Gastroenterology / hepatology** | | | | | | |
| *Candidiasis, oesophageal* | 4 | 3 (75) | 1 (25) | 3 | 3 (100) | 1 (33) |
| *Cryptosporidiosis diarrhoea, >1 month* | 6 | 4 (67) | 0 | 7 | 7 (100) | 4 (57) |
| *Isosporiasis, >1 month* | 4 | 2 (50) | 0 | 5 | 4 (80) | 3 (60) |
| Anal cancer/dysplasia | 6 | 6 (100) | 6 (100) | 12 | 10 (83) | 5 (42) |
| Hepatitis A | 6 | 1 (17) | 1 (17) | 7 | 2 (29) | 1 (14) |
| Hepatitis B (acute or chronic) | 11 | 11 (100) | 8 (73) | 17 | 13 (76) | 11 (65) |
| Hepatitis C (acute or chronic) | 9 | 6 | 5 | 16 | 14 | 11 |

| | | | | | | |
|---|---|---|---|---|---|---|
| | | (67) | (56) | | (88) | (69) |
| Unexplained chronic diarrhoea | 3 | 1 (33) | 0 | 4 | 3 (75) | 1 (25) |
| **Subtotal** | **49** | **34 (71)** | **21 (44)** | **71** | **56 (78)** | **37 (54)** |
| **Gynaecology / obstetrics** | | | | | | |
| *Cervical cancer* | 11 | 1 (9) | 0 | 14 | 7 (50) | 2 (14) |
| Cervical dysplasia | 10 | 2 (20) | 0 | 14 | 9 (64) | 3 (21) |
| Pregnancy (implications for the unborn child) | 6 | 5 (83) | 5 (83) | 25 | 24 (96) | 24 (96) |
| **Subtotal** | **27** | **8 (30)** | **5 (19)** | **53** | **40 (75)** | **29 (55)** |
| **Haematology** | | | | | | |
| *Non-Hodgkin lymphoma* | 11 | 11 (100) | 11 (100) | 11 | 11 (100) | 10 (91) |
| Castleman's disease | 1 | 1 (100) | 1 (100) | 3 | 2 (67) | 1 (33) |
| Idiopathic/thrombotic thrombocytopenic purpura | 6 | 5 (83) | 4 (67) | 13 | 10 (77) | 10 (77) |
| Malignant lymphoma/Hodgkin's lymphoma | 13 | 7 (54) | 6 (46) | 23 | 18 (78) | 18 (78) |
| **Subtotal** | **31** | **24 (77)** | **22 (71)** | **50** | **41 (82)** | **39 (78)** |
| **Internal Medicine** | | | | | | |
| *Atypical disseminated leishmaniasis* | 5 | 4 (80) | 1 (20) | 6 | 4 (67) | 3 (50) |
| *Candidiasis, bronchial/tracheal/lungs* | 3 | 0 | 0 | 4 | 3 (75) | 1 (25) |
| *Coccidioidomycosis, disseminated/extrapulmonary* | 1 | 1 (100) | 0 | 1 | 1 (100) | 1 (100) |
| *Cryptococcosis, extrapulmonary* | 3 | 3 (100) | 1 (33) | 2 | 1 (50) | 1 (50) |
| *Cytomegalovirus, other (except liver, spleen, glands)* | 3 | 2 (67) | 0 | 5 | 4 (80) | 2 (40) |

| | | | | | | |
|---|---|---|---|---|---|---|
| *Histoplasmosis, disseminated/extrapulmonary* | 1 | 0 | 0 | 1 | 1 (100) | 1 (100) |
| *Penicilliosis, disseminated* | 0 | 0 | 0 | 0 | 0 | 0 |
| *Reactivation of American trypanosomiasis (meningoencephalitis or myocarditis)* | 1 | 0 | 0 | 1 | 0 | 0 |
| *Salmonella septicaemia, recurrent* | 4 | 1 (25) | 0 | 5 | 4 (80) | 2 (40) |
| Candidaemia | 2 | 1 (50) | 0 | 3 | 0 | 0 |
| Candidiasis | 3 | 1 (33) | 0 | 8 | 4 (50) | 1 (13) |
| Invasive pneumococcal disease | 6 | 2 (33) | 0 | 1 | 1 (100) | 0 |
| Mononucleosis-like illness | 4 | 4 (100) | 1 (25) | 4 | 4 (100) | 4 (100) |
| Oral hairy leukoplakia | 3 | 1 (33) | 0 | 5 | 3 (60) | 3 (60) |
| Unexplained chronic renal impairment | 6 | 0 | 0 | 13 | 5 (38) | 3 (60) |
| Unexplained fever | 2 | 2 (100) | 2 (100) | 1 | 1 (100) | 1 (100) |
| Unexplained leukocytopenia/thrombocytopenia lasting >4 weeks | 6 | 6 (100) | 5 (83) | 4 | 3 (75) | 3 (75) |
| Unexplained lymphadenopathy | 4 | 2 (50) | 2 (50) | 5 | 4 (80) | 4 (80) |
| Unexplained oral candidiasis | 3 | 1 (33) | 0 | 5 | 3 (60) | 2 (40) |
| Unexplained weight loss | 2 | 1 (50) | 0 | 2 | 1 (50) | 1 (50) |
| Visceral leishmaniasis | 1 | 1 (100) | 0 | 5 | 4 (80) | 2 (40) |
| **Subtotal** | **63** | **33 (53)** | **13 (21)** | **81** | **51 (63)** | **37 (46)** |
| **Neurology / neurosurgery** | | | | | | |
| *Cerebral toxoplasmosis* | 4 | 3 (75) | 1 (25) | 5 | 5 (100) | 2 (40) |
| *Primary cerebral lymphoma* | 3 | 3 (100) | 3 (100) | 10 | 9 (90) | 7 (70) |

| | | | | | | |
|---|---|---|---|---|---|---|
| *Progressive multifocal leukoencephalopathy* | 0 | 0 | 0 | 2 | 1 (50) | 1 (50) |
| Cerebral abscess | 2 | 2 (100) | 2 (100) | 7 | 3 (43) | 2 (29) |
| Guillain–Barré syndrome | 3 | 2 (67) | 1 (33) | 4 | 3 (75) | 3 (75) |
| Lymphocytic meningitis | 4 | 3 (75) | 1 (25) | 6 | 4 (67) | 3 (50) |
| Mononeuritis | 3 | 2 (67) | 2 (67) | 4 | 3 (75) | 2 (50) |
| Multiple sclerosis-like disease | 5 | 3 (60) | 1 (20) | 9 | 5 (56) | 2 (22) |
| Peripheral neuropathy | 4 | 3 (75) | 1 (25) | 8 | 6 (75) | 1 (13) |
| Primary space occupying lesion of the brain | 0 | 0 | 0 | 3 | 2 (67) | 2 (67) |
| Subcortical dementia | 8 | 5 (63) | 2 (25) | 6 | 5 (83) | 4 (67) |
| **Subtotal** | **36** | **26 (72)** | **14 (39)** | **64** | **46 (72)** | **29 (45)** |
| **Ophthalmology** | | | | | | |
| *Cytomegalovirus retinitis* | 4 | 3 (75) | 0 | 5 | 5 (100) | 3 (60) |
| Infective retinal diseases, including herpes viruses and toxoplasma | 1 | 0 | 0 | 5 | 2 (40) | 1 (20) |
| **Subtotal** | **5** | **3 (60)** | **0** | **10** | **7 (70)** | **4 (40)** |
| **Pulmonology** | | | | | | |
| *Mycobacterium avium complex or Mycobacterium kansasii, disseminated or extrapulmonary* | 2 | 1 (50) | 1 (50) | 5 | 5 (100) | 3 (60) |
| *Mycobacterium, other species or unidentified species, disseminated or extrapulmonary* | 1 | 1 (100) | 1 (100) | 2 | 1 (50) | 1 (50) |
| *Mycobacterium tuberculosis, pulmonary or extrapulmonary* | 11 | 9 (82) | 8 (73) | 20 | 18 (90) | 10 (50) |
| *Pneumocystis carinii pneumonia* | 3 | 1 (33) | 1 (33) | 6 | 6 (100) | 3 (50) |
| *Pneumonia, recurrent (2 or more episodes in 12 months)* | 7 | 1 (14) | 1 (14) | 7 | 4 (57) | 1 (14) |

| | | | | | | |
|---|---|---|---|---|---|---|
| Community-acquired pneumonia | 6 | 1<br>(17) | 1<br>(17) | 12 | 7<br>(58) | 1<br>(8) |
| Primary lung cancer | 8 | 0 | 0 | 19 | 5<br>(26) | 3<br>(16) |
| **Subtotal** | **38** | **14<br>(37)** | **13<br>(34)** | **71** | **46<br>(65)** | **22<br>(31)** |

HIV = human immunodeficiency virus

**Supplementary Table S9: The total number and proportions of HIV indicator condition guidelines identified that report the association with HIV and recommend HIV testing per specialty overall, geographically ordered (Western and Eastern Europe ) and according to achieved 90-90-90 goals.**

| Specialty | Number of guidelines identified | Association with HIV reported n (%) | HIV testing recommended n (%) |
|---|---|---|---|
| **Dermatology / venereology** | | | |
| Total | 142 | 116 (82) | 81 (57) |
| Western Europe | 88 | 74 (84) | 47 (53) |
| Eastern Europe | 54 | 42 (78) | 34 (63) |
| 90-90-90 goals achieved | 49 | 38 (78) | 24 (49) |
| 90-90-90 goals not yet achieved | 93 | 78 (84) | 57 (61) |
| **Gastroenterology / hepatology** | | | |
| Total | 120 | 90 (75) | 58 (48) |
| Western Europe | 80 | 59 (74) | 39 (49) |
| Eastern Europe | 40 | 31 (78) | 19 (48) |
| 90-90-90 goals achieved | 49 | 34 (69) | 21 (43) |
| 90-90-90 goals not yet achieved | 71 | 56 (79) | 37 (52) |
| **Gynaecology / obstetrics** | | | |
| Total | 80 | 48 (60) | 34 (43) |
| Western Europe | 43 | 18 (42) | 9 (21) |
| Eastern Europe | 37 | 30 (81) | 25 (68) |
| 90-90-90 achieved | 27 | 8 | 5 |

| | | | |
|---|---|---|---|
| | | (30) | (19) |
| 90-90-90 not yet achieved | 53 | 40 (75) | 29 (55) |
| **Haematology** | | | |
| Total | 81 | 65 (80) | 61 (75) |
| Western Europe | 57 | 46 (81) | 44 (77) |
| Eastern Europe | 24 | 19 (79) | 17 (71) |
| 90-90-90 goals achieved | 31 | 24 (77) | 22 (71) |
| 90-90-90 goals not yet achieved | 50 | 41 (82) | 39 (78) |
| **Internal medicine** | | | |
| Total | 144 | 84 (58) | 50 (35) |
| Western Europe | 96 | 50 (52) | 23 (23) |
| Eastern Europe | 48 | 34 (71) | 27 (56) |
| 90-90-90 goals achieved | 63 | 33 (52) | 13 (21) |
| 90-90-90 goals not yet achieved | 81 | 51 (63) | 37 (46) |
| **Neurology / neurosurgery** | | | |
| Total | 100 | 72 (72) | 43 (43) |
| Western Europe | 70 | 49 (70) | 28 (40) |
| Eastern Europe | 30 | 23 (77) | 15 (50) |
| 90-90-90 goals achieved | 36 | 26 (72) | 14 (39) |
| 90-90-90 goals not yet achieved | 64 | 46 (72) | 29 (45) |
| **Ophthalmology** | | | |

| | | | |
|---|---|---|---|
| Total | 15 | 10 (67) | 4 (27) |
| Western Europe | 6 | 4 (67) | 1 (17) |
| Eastern Europe | 9 | 6 (67) | 3 (33) |
| 90-90-90 achieved | 5 | 3 (60) | 0 |
| 90-90-90 not yet achieved | 10 | 7 (70) | 4 (40) |
| **Pulmonology** | | | |
| Total | 109 | 60 (55) | 35 (32) |
| Western Europe | 63 | 29 (46) | 21 (33) |
| Eastern Europe | 46 | 31 (67) | 14 (30) |
| 90-90-90 goals achieved | 38 | 14 (37) | 13 (34) |
| 90-90-90 goals not yet achieved | 71 | 46 (65) | 22 (31) |

HIV = human immunodeficiency virus
